# Reasons for discarding blood from blood bank of government medical college, Aurangabad

**DOI:** 10.4103/0973-6247.76009

**Published:** 2011-01

**Authors:** Meenal M. Thakare, Jagannath V. Dixit, Naveen K. Goel

**Affiliations:** Department of Community Medicine, Government Medical College and Hospital, Chandigarh, India; 1 Department of Community Medicine, Government Medical College and Hospital, Aurangabad, India

Sir,

In this study we reviewed records of discarded blood from blood bank maintained by the Department of Pathology for three years i.e. 2005 to 2007. A total of 24,547 blood units were collected from blood donors during the period of three years. All donor blood samples were screened for HBV, HCV, HIV, and VDRL. On an average, 3.58% blood units were discarded over this time period. The main reason for discarding these blood units was the positivity for different Transfusion Transmissible Infections (TTIs), 68.86%, followed by other reasons (31.13%). Among the units discarded, 49.82% were positive for HBsAg, 10% for HIV, 8.97% for HCV, while no unit was positive for VDRL. About 31.13% units were discarded for other reasons. No unit was found with multiple infections.

In a study from Delhi, authors found a decreasing trend in the prevalence of all major TTIs over the past four years. The prevalence in our study was slightly lower than the abovementioned study in donors from the capital.[1] The prevalence of HIV, HBV, HCV, and VDRL was higher i.e. 0.32, 2.26, 0.23, and 0.45%, respectively, in Kolkata in the year 2002[2] than 0.35, 1.78, 0.32 and 0%, respectively, in Aurangabad in the year 2007.

Blood safety still depends highly on open and honest answering of the screening questions. Time has come when serious, concerted, and coordinated efforts with international support are required to strengthen blood transfusion services in a developing country like ours of Southeast Asian Region.
